# Germline mutations in *PPP2R1B* in patients with a personal and family history of cancer

**DOI:** 10.1172/jci.insight.186288

**Published:** 2025-04-03

**Authors:** Sahar Mazhar, Caitlin M. O’Connor, Alexis Harold, Amanda C. Dowdican, Peter J. Ulintz, Erika N. Hanson, Yuping Zhang, Michelle F. Jacobs, Sofia D. Merajver, Mark W. Jackson, Anthony Scott, Anieta M. Sieuwerts, Arul M. Chinnaiyan, Goutham Narla

**Affiliations:** 1 Division of Genetic Medicine, Department of Internal Medicine, Michigan Medicine, University of Michigan, Ann Arbor, Michigan, USA.; 2 Department of Pathology, Case Western Reserve University, Cleveland, Ohio. USA.; 3 BRCF Bioinformatics Core,; 4 Michigan Center for Translational Pathology, Michigan Medicine, and; 5 Division of Hematology and Oncology, Department of Internal Medicine, Michigan Medicine, University of Michigan, Ann Arbor, Michigan, USA.; 6 Department of Medical Oncology, Erasmus MC Cancer Institute, Rotterdam, Netherlands.

**Keywords:** Genetics, Oncology, Breast cancer, Genetic diseases, Tumor suppressors

## Abstract

An estimated 5%–10% of cancer results from an underlying genetic predisposition. For the majority of familial cases, the genes in question remain unknown, suggesting a critical need to identify new cancer predisposition genes. Members of the protein phosphatase 2A (PP2A) family exist as trimeric holoenzymes and are vital negative regulators of multiple oncogenic pathways. PP2A inhibition by somatic mutation, loss of expression, and upregulation of its exogenous inhibitors in tumors has been well described. However, it remains unknown whether germline loss of any PP2A subunits results in a predisposition to cancer in humans. In this study, we identified 9 cancer patients with germline loss-of-function (LOF) variants in *PPP2R1B* (Aβ), the β isoform of the PP2A scaffold subunit. All 4 patients for whom documentation was available also had a family history of cancer, including multiple indicators of hereditary cancer. Overexpression of these mutant forms of Aβ resulted in truncated proteins that were rapidly turned over. Characterization of an additional missense germline Aβ variant, R233C, which is also recurrently mutated at the somatic level, showed disruption of PP2A catalytic subunit binding, resulting in loss of phosphatase activity. An analysis of Aβ expression among multiple breast cancer cohorts (the most highly represented cancer among the Aβ germline patients) revealed that somatic, heterozygous loss of Aβ was a frequent event in this disease, and decreased Aβ expression correlated with shorter disease-free and overall survival. Furthermore, Aβ levels were significantly lower in multiple histological subtypes of both in situ and malignant breast cancer compared with adjacent normal breast tissue, suggesting that Aβ loss is an early event in breast cancer development. Together, these results highlight a role for Aβ as a predisposition gene in breast cancer and potentially additional cancers.

## Abstract 2

<a>We identified patients with germline loss of function variants in *PPP2R1B* with family histories of cancer and describe a role for *PPP2R1B* as a predisposition gene for breast cancer.</a>

## Introduction

Protein phosphatase 2A (PP2A) belongs to a serine/threonine family of phosphatases that primarily exist as heterotrimeric holoenzymes (1). Synthesis of this complex is initiated by the formation of an AC dimer, consisting of a scaffold subunit (Aα or Aβ) and a catalytic subunit (Cα or Cβ) (2). Formation of the initial dimer allows the recruitment of one of a diverse set of regulatory (B) subunits. About 15 different genes code for B subunits, which have been divided into 4 families (PR130/PR72, B56, B55, and Striatins) (3–6). B subunits dictate the substrate specificity of the holoenzyme (7–9).

PP2A has multiple roles in development, cell cycle regulation, cell survival, and apoptosis, and all 3 components of the trimeric holoenzyme have been implicated as tumor suppressors (10–13). Perhaps best characterized among these is the Aα scaffold, which is found to be mutated in up to 30% of serous endometrial tumors, and hotspot mutations of this gene result in loss of B subunit recruitment and tumor suppressive function (14, 15). Cellular models of transformation have shown that suppression of PP2A is a critical step in oncogenic progression (16, 17). Coexpression of the SV40 large T antigen, telomerase catalytic subunit, and mutant HRAS in epithelial cells requires an additional step involving the inhibition of at least one PP2A subunit such as Cα, Aα, Aβ, or one of the tumor suppressive B subunits to result in complete transformation, as characterized by the ability to grow in an anchorage-independent manner in vitro and to form tumors in immunocompromised mice (18–20). Intriguingly, while Aα and Aβ are 86% identical in their amino acid sequence, the transformation program driven by Aβ suppression cannot be compensated by the predominant scaffold, Aα. This suggests that holoenzymes composed of the Aβ scaffold have unique tumor suppressive properties independent of Aα (19).

Multiple studies have proposed a role for Aβ suppression in cancer progression. Dysregulated splicing of Aβ leading to truncated transcripts and decreased expression has been reported in hepatocellular carcinoma and B cell leukemia (21, 22). Among PP2A family members, Aβ was the only subunit found to be significantly decreased in a cohort of acute myeloid leukemia samples compared with normal CD34^+^ cells (23). In colon cancer, microRNA-mediated inhibition of Aβ drives resistance to fluorouracil (5-FU) treatment by eliminating Aβ-directed dephosphorylation of AKT (24, 25). Restoration of Aβ resensitizes these cells to 5-FU in an AKT-dependent manner. In addition, a large-scale genome-wide association study, coupled with transcriptomic data, identified a breast cancer susceptibility locus upstream of the Aβ gene (26). Previous groups have reported germline missense variants in cancer patients, the most common being G90D (27), but the functional effect of this change on the Aβ protein, if any, remains unclear.

Approximately 5%–10% of human cancer results from inherited germline mutations in tumor suppressor genes or oncogenes (28). Germline mutations in PP2A subunits Aα, Cα, B56β, B56γ, and B56δ have been identified in patients with intellectual disability (29–31), but so far, germline mutations in PP2A family members have not been linked to cancer predisposition. In this study, we report multiple cancer patients with truncating, loss-of-function (LOF) (28) germline mutations in the Aβ gene that were identified by the Michigan Oncology Sequencing Center (MI-ONCOSEQ) (32). Many of these patients have a family history of cancer, further suggesting an inherited component of the disease. Overexpression of the truncated forms of Aβ revealed that the mutant Aβ is likely targeted for proteasomal degradation. An additional missense variant, R233C identified in a breast cancer patient, was also characterized as a LOF change, as it prevents catalytic subunit binding. Analysis of Aβ levels in multiple breast cancer cohorts revealed that heterozygous loss of the Aβ gene and decreased mRNA expression are common events in breast cancer development, suggesting that monoallelic, germline, LOF variants in this gene may result in a predisposition to cancer.

## Results

### Large-scale sequencing efforts identify multiple cancer patients with deleterious germline variants in PPP2R1B.

**Figure 1:**
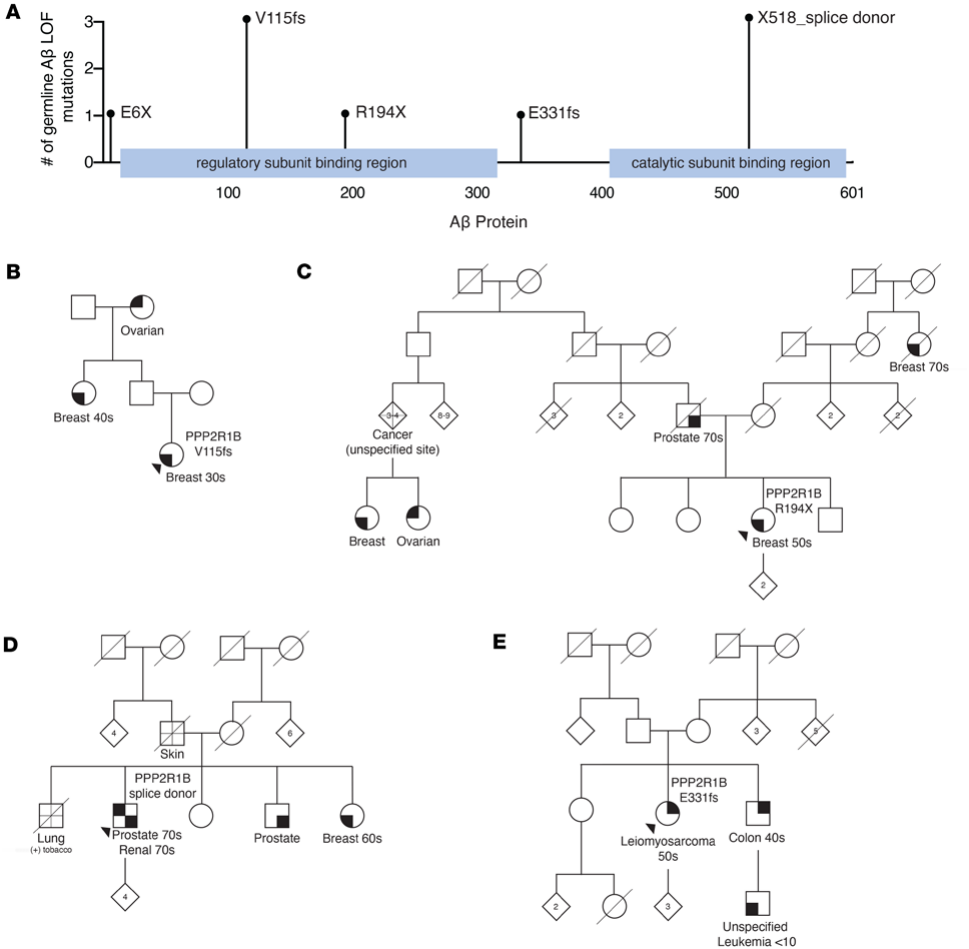
Multiple cancer patients identified with germline loss-of-function *PPP2R1B* (Aβ) mutations and a family history of cancer. (**A**) Location of truncating Aβ germline mutations on a schematic of the Aβ protein with regulatory and catalytic subunit binding sites highlighted. (**B**–**E**) Pedigrees demonstrating a family history of cancer in patients with germline Aβ mutations.

The MI-ONCOSEQ has somatic and germline sequencing data for over 4000 cancer patients (32). We queried this database for patients harboring LOF germline variants in tumor suppressive PP2A subunits. Eight patients in this cohort contain LOF (presumed deleterious) mutations in the gene *PPP2R1B* (Aβ), the β isoform of the scaffold subunit (Table 1). Given that 3 of the patients had breast cancer, we also queried for Aβ LOF germline variants in the breast cancer cohort of The Cancer Genome Atlas (TCGA), where we found an additional patient with the V115 frameshift mutation (Table 1). The locations of the identified mutations on the Aβ protein relative to the sites of regulatory and catalytic subunit binding are shown in Figure 1A. The most C-terminal mutation is a splice donor LOF variant at the end of exon 12 (Figure 1A). The C-terminal end of the Aβ protein (HEAT repeats 11–15, aa positions 412–601) is the region that binds the PP2Ac catalytic subunit (33). As a result, these truncating mutations are predicted to lose PP2Ac binding. The identified mutations occur at a very low frequency in the general population (Supplemental Table 1; supplemental material available online with this article; https://doi.org/10.1172/jci.insight.186288DS1), the most common being the V115 frameshift mutation that occurs in 1 in 550 individuals in the European population, while E6X and E331fs have never been previously reported in any population (34). To determine whether additional truncating mutations in Aβ have been identified in the general population, we looked at all Aβ germline variants annotated as LOF in gnomAD (34) (Supplemental Figure 1). All annotated Aβ LOF variants other than V115fs are rare, with less than 0.01% allele frequency.

Among the patients identified with Aβ germline mutations, additional information regarding their family history of cancer was available for 4 patients (Figure 1, B–E). Patient 4 has a germline Aβ V115fs variant and has a personal history of breast cancer diagnosed in her thirties. In addition, her paternal grandmother and paternal aunt have a history of ovarian and breast cancer, respectively. Patient 5, with a germline Aβ R194X variant, has a personal history of breast cancer diagnosed in her 50s and a family history of breast, ovarian, and prostate cancer. Patient 6 has a personal history of uterine leiomyosarcoma diagnosed in her 50s. Additionally, her brother was diagnosed with colon cancer, and she has a nephew (from the same brother) with a history of childhood leukemia. Patient 7 contains a germline LOF variant at the Aβ exon 12 splice donor site that destroys the conserved splice site and is predicted to result in either intron retention and truncation or exon skipping. He has a personal history of multiple types of cancer, including prostate and renal cancer. In addition, he has 3 siblings with a history of cancer, including breast and prostate cancer.

All 4 families demonstrate a potential cancer predisposition phenotype based on the age of diagnoses, cancer types, and/or number of individuals with cancers, despite lacking pathogenic variants in established predisposition genes (Supplemental Table 2). Given the tumor suppressive role of Aβ, we further investigated the role of these variants in Aβ function.

### Breast cancer–derived LOF germline variants in Aβ result in truncated Aβ products that are rapidly turned over.

**Figure 2:**
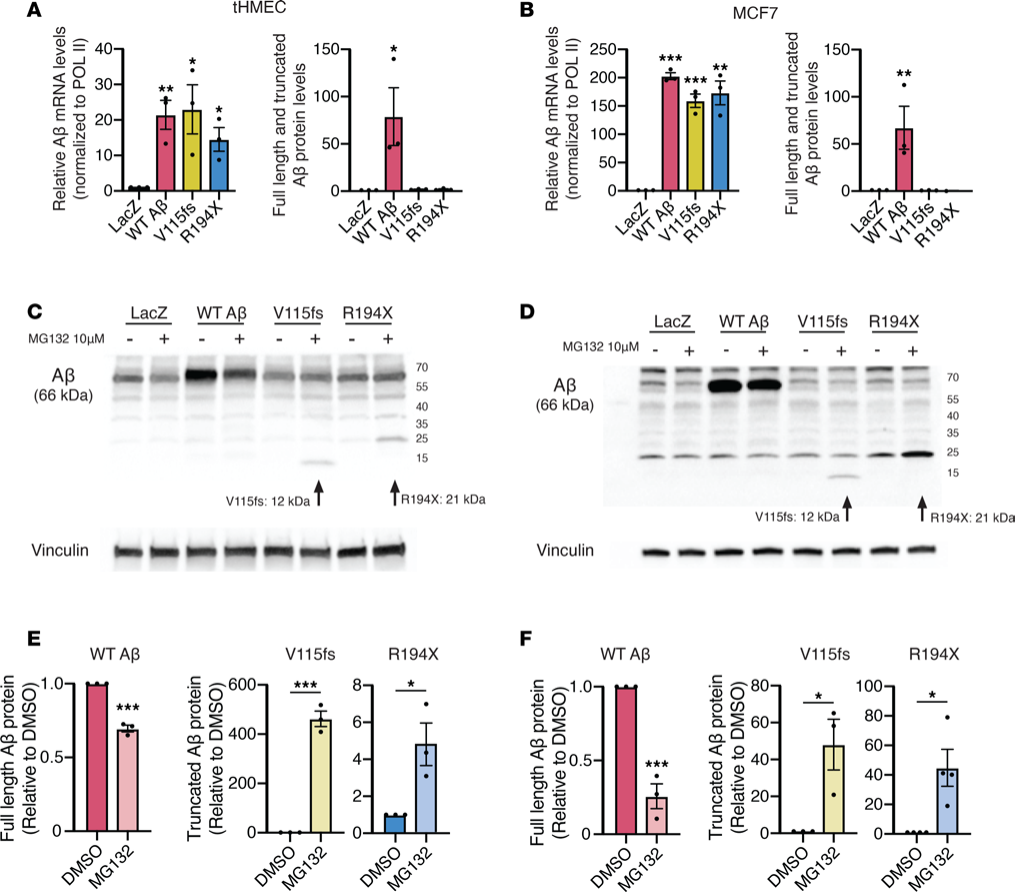
Breast cancer–derived Aβ germline truncating mutants are targeted for proteasomal degradation. Quantitation of overexpression of wild-type and mutant Aβ mRNA and protein in (**A**) tHMECs and (**B**) MCF7 cells. Western blots for Aβ in wild-type and mutant-expressing cell lines in (**C**) tHMECs and (**D**) MCF7 cells treated with MG-132. Quantitation of wild-type and mutant forms of Aβ in (**E**) tHMECs and (**F**) MCF7 cells treated with MG-132. Blots are representative of 3 experiments. Data are presented as mean ± SEM, *n* = 3–4. *P < 0.05; **P < 0.01; ***P < 0.001 by 2-tailed Student’s *t* test.

The PP2Ac catalytic subunit is predicted to bind Aβ on its C-terminal HEAT repeats 11–15, suggesting that truncation mutations along this region or before this region will result in loss of Aβ catalytic activity. To determine whether the germline Aβ LOF variants V115fs and R194X identified in patients with breast cancer result in truncated Aβ proteins that lose catalytic subunit binding, wild-type or truncated forms of Aβ were overexpressed in 2 breast cancer cell lines. In both RAS-transformed human mammary epithelial cells (tHMECs) and MCF7 cells, overexpression was successfully achieved at the mRNA level (Figure 2, A and B). However, exogenous Aβ protein was only detected in the cell lines overexpressing wild-type Aβ, suggesting that the truncated forms of Aβ may be targeted for degradation. Treatment with the proteasomal inhibitor MG-132 recovered the truncated forms of Aβ in both tHMECs and MCF7 cells (Figure 2, C–F). Oddly, wild-type Aβ was not further stabilized by MG132 treatment, and wild-type protein decreased with proteasome inhibition. To demonstrate that MG-132 was successfully inhibiting the proteasome, stabilization of the short-lived proteins p53 and cyclin D3 was also shown (Supplemental Figure 2). Given that the germline variants V115fs and R194X result in highly labile, truncated forms of Aβ, we hypothesized that these mutations were functionally equivalent to heterozygous loss of Aβ. To determine whether the remaining wild-type allele was inactivated at the somatic level, we queried RNA-seq data from MI-ONCOSEQ and TCGA to see whether Aβ levels were decreased in tumors of patients with germline truncating mutations. To compare relative mRNA levels, the MI-ONCOSEQ or TCGA breast cohort was divided into 3 groups for *PPP2R1B* expression: Low (<25th percentile), Normal (25th< and >75th percentile), and High (>75th percentile). For most patients, the Aβ tumor allele frequency is around 50%, suggesting that the wild-type allele is still present. However, only 3 out of 9 Aβ germline patients have low levels of Aβ mRNA, suggesting that in most cases, type Aβ mRNA is still present in tumors.

### A recurrent, somatic Aβ mutation also occurs in the germline of a breast cancer patient and loses catalytic subunit binding.

**Figure 3:**
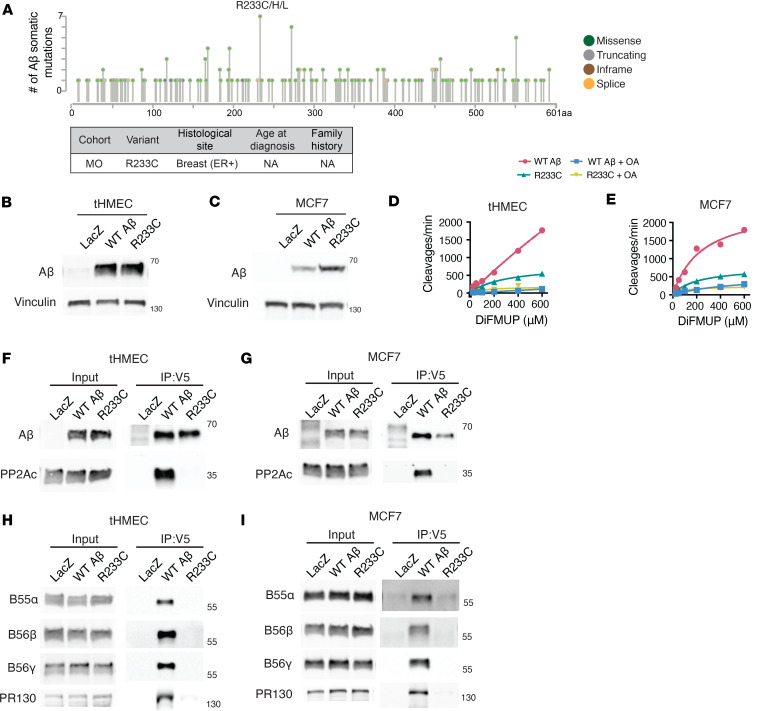
The recurrent Aβ somatic mutation R233C/H/L was discovered in the germline of a patient with breast cancer and causes loss of holoenzyme assembly. (**A**) Representation of all somatic mutations in Aβ reported from over 100,000 patients across 226 studies in the cBioPortal database. Somatic missense mutations at R233 are reported in 7 patients in cBioPortal and in 1 patient at the germline in the MI-ONCOSEQ cohort. (**B** and **C**) Western blots for whole-cell lysates from tHMECs and MCF7 cells overexpressing V5-tagged control LacZ, wild-type Aβ, and Aβ-R233C mutant. (**D** and **E**) Phosphatase activity assays against V5 Co-IP from wild-type or R233C Aβ–expressing (**D**) tHMECs and (**E**) MCF7 cells. Okadaic acid (OA) is a catalytic inhibitor of PP2Ac and was used as a negative control. Experiments were conducted 3 times. (**F** and **G**) Co-IP immunoblots for PP2Ac catalytic subunit bound to wild-type or R233C forms of Aβ in (**F**) tHMECs or (**G**) MCF7 cells. (**H** and **I**) CoIP immunoblots for B regulatory subunits bound to wild-type or R233C forms of Aβ in (**H**) tHMECs or (**I**) MCF7 cells. Data are representative of 3 experiments.

Somatic mutations in Aβ are relatively rare. We queried Aβ somatic mutations across all 226 studies in cBioPortal (35, 36), representing over 100,000 patients. We found that 199 patients contain a somatic change in Aβ, with the vast majority being missense mutations (Figure 3A). The most recurrent somatic missense mutation is a change at R233, reported in 7 patients (Figure 3A and Supplemental Table 4). The R233C mutation was also discovered in the germline of a single estrogen receptor–positive (ER^+^) breast cancer patient in the MI-ONCOSEQ cohort. Since this particular variant was found in both the somatic and germline settings, additional functional studies were performed to determine whether it resulted in LOF of Aβ. tHMECs and MCF7 cells overexpressing V5-tagged Aβ-R233C were generated (Figure 3, B and C). To determine whether the Aβ-R233C variant formed catalytically active complexes, coimmunoprecipitation (Co-IP) of exogenously expressed wild-type or mutant Aβ was performed using an anti-V5 antibody. The phosphatase activity of the V5-bound complex was determined against the fluorescent substrate DiFMUP. The addition of okadaic acid, a potent catalytic site inhibitor of PP2A (37), was used as a negative control. In both cell lines, the R233C-bound complexes had markedly reduced catalytic activity (Figure 3, D and E, and Supplemental Figure 3A). To determine whether the introduction of the R233C cancer-derived point mutant was disrupting catalytic subunit binding, the V5-bound Co-IP was analyzed by Western blotting for the PP2Ac catalytic subunit. In both tHMECs and MCF7 cells, the R233C mutant resulted in over 90% loss of catalytic subunit binding (Figure 3, F and G, and Supplemental Figure 3B). Additionally, there was a substantial decrease in binding of multiple regulatory subunits (Figure 3, H and I, and Supplemental Figure 3C), consistent with the model of PP2A heterotrimer assembly, whereby the A-C dimer must form first in order for B regulatory subunits to bind. These functional studies suggest that in addition to germline truncating mutations, germline missense mutations in Aβ, such as R233C, may contribute to the loss of Aβ tumor suppressive ability through loss of catalytic and regulatory subunit binding.

### Aβ copy number and mRNA are decreased in breast cancer, and lower Aβ levels are correlated with more aggressive disease and reduced survival.

**Figure 4:**
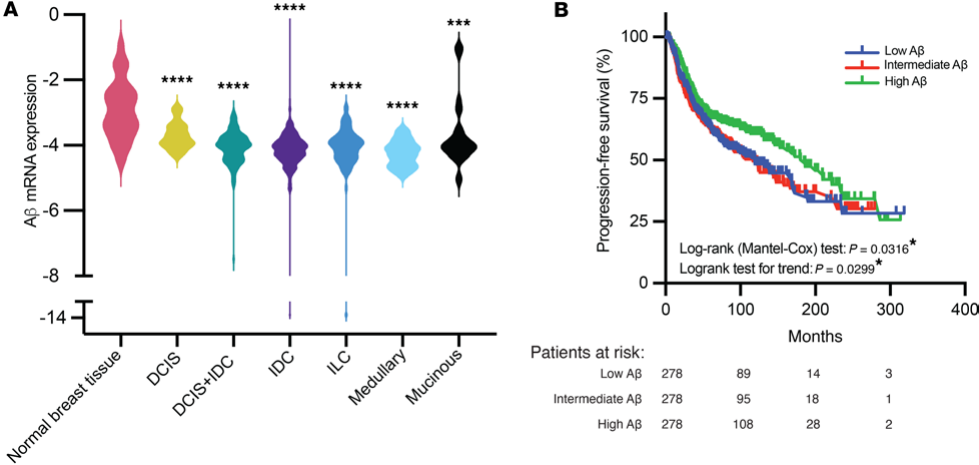
Decreased Aβ mRNA in breast cancer correlates with reduced disease-free survival. (**A**) qRT-PCR for Aβ in normal and histologically diverse breast cancer samples from patients who did not have evidence of lymph node or distant metastasis. Aβ mRNA expression is expressed as the natural log of the ΔCt ratio of Aβ and reference genes. Significance was calculated using a 1-way ANOVA with Dunnett’s multiple-comparison test. ***P < 0.001, ****P < 0.0001. (**B**) Kaplan-Meier analysis of disease-free progression of lymph node–negative patients. The cohort was trichotomized into 3 groups based on Aβ expression. DCIS, ductal carcinoma in situ; ILC, invasive lobular carcinoma; IDC, invasive ductal carcinoma.

Since cancer predisposition genes such as BRCA1/2 are often lost at the somatic level in tumors (38), even in patients without germline mutations, we looked at mRNA levels of Aβ in normal breast tissue and from lymph node–negative breast cancer patient samples in the tumor bank at Erasmus University Medical Center. Aβ levels were significantly decreased in all breast cancer histological subtypes compared with normal adjacent breast tissue (Figure 4A). Additional clinical characteristics, where available, are represented in Supplemental Table 5. There were no significant associations between Aβ expression levels and ER status, age at primary surgery, or menopause status. Aβ was significantly lower in tumors lacking progesterone receptor (PR) and in tumors that were ERBB2 (HER2) negative. Furthermore, Aβ expression was significantly decreased in poorly differentiated tumors (classified by pathologists as poor grade), and tumors with lower Aβ were associated with reduced disease-free survival (Figure 4B and Supplemental Table 5). To determine whether Aβ mRNA loss in tumors could be explained by Aβ genomic deletion, we looked at publicly available databases for which Aβ copy number and mRNA data were available. A pan-cancer analysis of Aβ copy number in TCGA demonstrated that breast cancer has one of the highest levels of Aβ heterozygous loss, with shallow deletions occurring in approximately 48% of breast cancers (Supplemental Figure 4A). Heterozygous loss of the Aβ gene was significantly associated with decreased Aβ mRNA (Supplemental Figure 4B). This is mirrored in another breast cancer cohort, the Molecular Taxonomy of Breast Cancer International Consortium (METABRIC) (39), where heterozygous loss was seen in 40% of patients (Supplemental Figure 4C) and similarly, this results in significantly reduced Aβ mRNA expression in this cohort (Supplemental Figure 4D). METABRIC is the largest publicly available cohort for which Aβ copy number, mRNA, and patient survival data exist. Both heterozygous loss of Aβ and lower Aβ mRNA levels were significantly associated with reduced overall survival in this cohort (Supplemental Figure 4, E and F). The loss of Aβ in breast cancer and its association with poor prognosis suggests a crucial tumor suppressive role for this gene in breast cancer, potentially affecting multiple stages in tumor development and recurrence.

### Trancriptomic analysis of tumors from Aβ germline LOF patients reveals enrichment of immune-related pathways.

To investigate altered signaling pathways downstream of Aβ loss using gene set enrichment analysis (GSEA), 16 control samples were selected from MI-ONCOSEQ tumor samples with no Aβ germline mutation detected. Since the Aβ germline tumors were from multiple cancer types (breast, prostate, kidney, multiple myeloma, leiomyosarcoma, and lymphoma), control samples were also selected from these cancer types. The top Hallmark pathways significantly altered in Aβ germline LOF tumors are shown in Supplemental Figure 5A. Both inflammatory response and IFN-γ response pathways were significantly enriched in Aβ-mutant tumors (Supplemental Figure 5B), suggesting these patients have a heightened tumor inflammation signature and may benefit from immunotherapy.

## Discussion

The tumor suppressive role of the PP2A family of phosphatases has been well established, with loss of PP2A activity reported across multiple cancer types and reactivation of PP2A being actively explored as a therapeutic strategy (40, 41). While inactivating, driver mutations in the predominant scaffold subunit Aα have been characterized (10, 14, 15, 42, 43), the scaffold subunit Aβ remains relatively understudied, despite early reports demonstrating that loss of Aβ contributes to oncogenic transformation of epithelial cells, facilitating their anchorage-independent growth and tumorigenicity (19).

Consistent with the hypothesis that Aβ loss may be an early event in cancer initiation, we identified 8 patients with germline LOF mutations in the Aβ gene *PPP2R1B* in a cohort of approximately 4000 cancer patients enrolled in the MI-ONCOSEQ study. Additional family history was available for 4 of these patients and revealed multiple indicators of potential familial cancer. Patient 4 harboring an Aβ V115fs variant was diagnosed with breast cancer at a young age in her thirties, and has 2 family members spanning a total of 3 generations diagnosed with breast or ovarian cancer. Based on this pedigree, the family meets criteria for germline evaluation of hereditary breast and ovarian cancer syndrome (HBOC) (44), but in the absence of any pathogenic variants in established predisposition genes in the proband, the familial cancer cannot be attributed to a particular gene. In such cases, since there is no way to definitively rule out an underlying genetic cause, relatives are presumed to have an elevated risk for breast and ovarian cancer, and may be recommended to undergo high-risk management based on family history. Both Patients 5 and 7 have a family history of breast and prostate cancer. Notably, Patient 7 has a personal history of multiple primary cancers, an additional indicator of an underlying predisposition. Finally, Patient 6 has a personal history of uterine leiomyosarcoma, a brother with a young diagnosis of colon cancer, and a nephew with history of childhood leukemia. This family would be a candidate for testing for Lynch syndrome (45), a condition characterized by familial colorectal and uterine cancers (predominantly epithelial, but sarcomas have also been reported; ref. 46), as well as other hereditary cancer predisposition genes. However, similar to the family of Patient 4, since there are no pathogenic variants in Lynch syndrome–associated genes, or other genes with known hereditary cancer risk, the cause of the elevated cancer risk in this family remains unknown.

Out of 8 cancer patients with Aβ LOF variants, 3 had breast cancer. Because of this, we applied for access to germline protected data for TCGA breast cancer cohort, and identified an additional cancer patient with a truncating variant in Aβ. Extrapolating from the crystal structure of Aα, with which Aβ shares 86% amino acid sequence, these truncating mutations are predicted to lose binding to the catalytic subunit (33). Attempts to overexpress the open-reading frames for *PPP2R1B* bearing either the V115fs or R194X mutations revealed that these truncated products were rapidly degraded by the proteasome. Although not tested here, it is also possible that the mutant transcripts are targeted by nonsense-mediated decay, as this is a common mechanism for clearing aberrant transcripts with premature stop codons (47).

In addition to the stop-gain and frameshift mutations described above, 3 cancer patients harbor identical splice donor variants at exon 12 (+1 site immediately following the end of exon 12). Splice donor (GU at +1 and +2) and splice acceptor (AG at –2 and –1) regions are extremely conserved and mutations lead to either intron retention or exon skipping (48). Aberrant splicing of Aβ leading to exon skipping and truncated transcripts has previously been reported in hepatocellular carcinoma and B cell leukemia (21, 22). However, whether these mutant transcripts are translated remains unknown. The presence of these truncated Aβ proteins may harbor dominant-negative effects by serving as a sink for certain Aβ-interacting partners, while losing the ability to form functional holoenzymes. Thus, follow-up work should determine whether these products are translated.

In addition to the 8 LOF germline variants in MI-ONCOSEQ, we observed multiple cancer patients with Aβ missense variants and proceeded to characterize the R233C germline variant since it is also a recurrent somatic change in cancer. The R233C mutation results in decreased phosphatase activity due to the inability to bind the catalytic subunit or any of the regulatory subunits investigated. Sablina et al. previously characterized several rare, somatic, cancer-derived mutations in Aβ and similarly found that they lost B regulatory and/or catalytic subunit binding to varying degrees (19). Combined, these observations suggest that cancer-derived point mutations in Aβ may lead to loss of tumor suppressive activity due to severely crippled catalytic function (as in the case of R233C) or as a result of skewed phosphatase activity against only a subset of substrates, as shown previously in the case of mutants like P65S that can bind the catalytic subunit, but not the tumor suppressive regulatory subunits B56α and B56γ.

Given the tumor suppressive roles of Aβ previously described, and the presence of germline LOF or deleterious mutations in at least 5 breast cancer patients thus far, we sought to determine whether loss of Aβ was a common event in breast cancer development. Analysis of Aβ mRNA levels in breast tumors revealed that Aβ was significantly decreased in all subtypes of breast cancer when compared with adjacent normal tissue. This analysis was performed in patients who were lymph node negative and did not receive any adjuvant systemic therapy. These patients went on to receive either breast-conserving surgery (55%) or modified mastectomy (45%), and 62% also received adjuvant radiotherapy. Kaplan-Meier analysis of disease-free progression tracked over a period of 3 years revealed that patients who had higher levels of Aβ prior to surgery had longer progression-free survival. This suggests that in addition to a role in cancer initiation, decreased Aβ may to contribute to breast cancer recurrence.

To determine whether the mechanism of Aβ loss in breast cancer originated at the genomic level, we looked at Aβ copy number in TCGA and METABRIC breast cancer cohorts. Indeed, heterozygous loss of the *PPP2R1B* gene is reported in 48% of breast tumors in TCGA and 40% of tumors in METABRIC. Since copy number loss is significantly associated with decreased mRNA levels in these cohorts, it is likely that the predominant mechanism of somatic Aβ loss in breast cancer is shallow deletion of the Aβ locus. Interestingly, biallelic deletion of Aβ is rarely observed, suggesting that while partial loss of Aβ is tumorigenic, complete loss of Aβ is actually disadvantageous to growth. This phenomenon has been well described for the closely related protein Aα (49), and was also previously shown in cellular models for the PP2A catalytic subunit (20).

Currently, multiple transgenic mouse models exist in which the knock-out or mutation of a specific PP2A subunit results in spontaneous tumor development or increased tumorigenesis in response to chemical or genetic insults. Homozygous loss of the B56δ regulatory subunit causes both hematological malignancies and hepatocellular carcinoma (50). Heterozygous deletion of exon 5–6 or the point mutant E64D in Aα enhances lung carcinogenesis induced by benzopyrene or oncogenic RAS (51, 52). Similar analysis of the Aβ heterozygous knockout mouse will be critical to determine whether germline loss of Aβ is sufficient to result in tumor development and further strengthen its role as a predisposition gene in cancer. Furthermore, cascade testing of family members of cancer patients harboring LOF Aβ mutations will further our understanding of how penetrant these changes are in terms of their predisposition to cancer and also allow the study of germline modifiers that influence this. Since the underlying genetic causes of 50% of familial breast cancers remain unknown (53), characterizing new predisposition genes will result in earlier detection of cancers due to enhanced screening of carriers, as well as pave the way to novel therapeutics from a better understanding of dysregulated signaling downstream of the germline event.

## Methods

### Sex as a biological variable.

Sex was not considered a biological variable in the study, as the Erasmus University cohort consists of only female breast cancer tissue.

### Cell culture.

Cells were grown at 37°C with 5% CO_2_ in a humidified incubator. MCF7 cells were obtained from ATCC and maintained in RPMI-1640 supplemented with 10% FBS, 0.5% penicillin/streptomycin, and 10 μg/mL insulin (Sigma-Aldrich, I0516). tHMECs (transformed by stable knockdown of TP53 and CDKN2A and overexpression of MYC and HRAS G12V) were supplied in-house and grown in modified MCDB170 media (54) consisting of a 1:1 ratio of Medium 171 (Thermo Fisher Scientific, M171500) and DMEM-F12 (GE Healthcare, SH30023.FS) supplemented with MEGS (Thermo Fisher Scientific, S0155), 0.5% penicillin/streptomycin, 5 μM isoproterenol (Sigma-Aldrich, 420355), 0.1 nM oxytocin (Bachem, 4016373), 0.5 ng/mL cholera toxin (Sigma-Aldrich, C8052), 5 nM tri-iodothyronine (Sigma-Aldrich, T2877), 0.25% FBS, 5 μg/mL insulin (Sigma-Aldrich, I0516), 0.5 nM β-estradiol (Sigma-Aldrich, E8875), 50 ng/mL hydrocortisone (Sigma-Aldrich, H4001), 2.5 ng/mL epidermal growth factor, 0.5× L-glutamine, 2.5 μg/mL transferrin (Sigma-Aldrich, T2252) and 0.1% Albumax (Thermo Fisher Scientific, 11021029). Wild-type– and mutant *PPP2R1B*–overexpressing lines were generated using lentiviral transduction as previously described (55). For MG-132 treatment, cells were treated with 10 μM MG-132 (Sigma-Aldrich, M7449) for 6–12 hours.

### Immunoblotting.

Cleared lysates (60 μg) were run in 12% or gradient (4%–15%) TGX Stain-free gels (Bio-Rad) and transferred to nitrocellulose membranes (Bio-Rad, 1704158). Proteins were detected using chemiluminescence (GE Healthcare, RPN2232) using the Bio-Rad Chemidoc XRS^+^. Antibodies used were against PPP2R1B (Aβ) (Aviva Systems Bio, OAAB1890, vinculin (Santa Cruz Biotechnology, sc-73614; V5-tag (for IP) (Bio-Rad, MCA1360GA), V5-tag (for immunoblotting) (Cell Signaling Technology, 13202S), PP2Ac (Abcam, ab106262), PP2A regulatory subunit Bα (B55α) (Santa Cruz Biotechnology, sc-81606), PP2A regulatory subunit Bβ (B56β) (Santa Cruz Biotechnology, sc-515676), PP2A regulatory subunit Bγ (B56γ) (Santa Cruz Biotechnology, sc-374380), PP2A regulatory subunit Bα (PR130) (Thermo Fisher Scientific, PA530127), cyclin D3 (Cell Signaling Technology, 2936S), and TP53 (Santa Cruz Biotechnology, sc-126).

### Co-IP and phosphatase assay.

Co-IP of V5-tagged wild-type and mutant *PPP2R1B* was performed according to the manufacturer’s protocol (Thermo Fisher Scientific, 14321D). Briefly, cells were lysed in phosphatase lysis buffer (25 mM HEPES, 1 mM MgCl_2_, 0.5% Triton X-100, and protease inhibitors) and 5 mg of lysate was loaded onto magnetic beads conjugated with anti-V5 antibody for 30 minutes. Unbound protein was washed away with phosphatase assay buffer (25 mM HEPES, 1 mM MgCl_2_, 0.1 mM MnCl_2_). Half of the beads were set aside for Western blotting and the remaining beads were used for phosphatase assays using the substrate DiFMUP (Thermo Fisher Scientific, D6567) as previously described (14), with the following modification: final activity was normalized to the amount of V5-tagged PPP2R1B (the bait) as determined by Western blotting.

### GSEA pathway analysis.

Differential analysis was performed with the Limma-voom approach (56, 57); cancer type was included in the model in addition to *PPP2R1B* mutation status to avoid confounding effects of various cancer types. Enrichment of Hallmark gene sets downloaded from MSigDB (58) were examined with fgsea (59) using genes ranked by logFC estimated from Limma as input.

### Erasmus University tumor biobank mRNA analysis.

*PPP2R1B* mRNA levels were determined using TaqMan and expressed as the natural log of the ΔCt ratio of *PPP2R1B* and reference genes *PBGD*, *HPRT*, and *B2M*.

### Statistics.

All statistical calculation were performed using GraphPad Prism version 8.3.1. Significance was calculated using a 2-tailed Student’s *t* test unless otherwise stated. A *P* value of less than 0.05 was considered significant.

### Study approval.

MI-ONCOSEQ sample collection and analysis*:* The study was approved under the University of Michigan Institutional Review Board (IRB) protocols HUM00046018, HUM00067928, and HUM00056496. Patients who were 18 years or older provided written informed consent for molecular profiling of tumor and normal tissue. Processing of tumor/germline tissue and clinical sequencing methods have been previously described (32).

Erasmus University tumor biobank: This retrospective study used coded freshly frozen primary tumor tissues of patients with primary operable breast cancer from 1978 through 2000 and was performed in accordance with the Medical Ethical Committee of the Erasmus Medical Center Rotterdam, The Netherlands (60). A protocol to study biological markers in tumor tissue that remained after surgical removal of the primary tumor was reviewed by the medical ethics committee of the Erasmus University Medical Center (MEC 02.953) and consent was not required in accordance with the Code of Conduct of the Federation of Medical Scientific Societies in The Netherlands. Patient enrollment criteria and tumor tissue quality control measures have been previously described (61).

### Data availability.

Values for all data points in graphs are reported in the Supporting Data Values file.

## Author contributions

SM and GN designed and conceptualized the study. SM, CMO, AH, ACD, PJU, ENH, AMS, and MFJ contributed to the acquisition of data. SM, CMO, AH, ACD, PJU, ENH, MFJ, SDM, MWJ, AS, AMS, AMC, and GN contributed to the analysis and interpretation. YZ contributed to acquisition, analysis and interpretation of data. MWJ provided tHMECs. SM wrote the original draft of the manuscript. All authors contributed to the manuscript editing and approved the submitted version.

## Supplementary Material

Supplemental data

Unedited blot and gel images

Supporting data values

## Figures and Tables

**Table 1:**
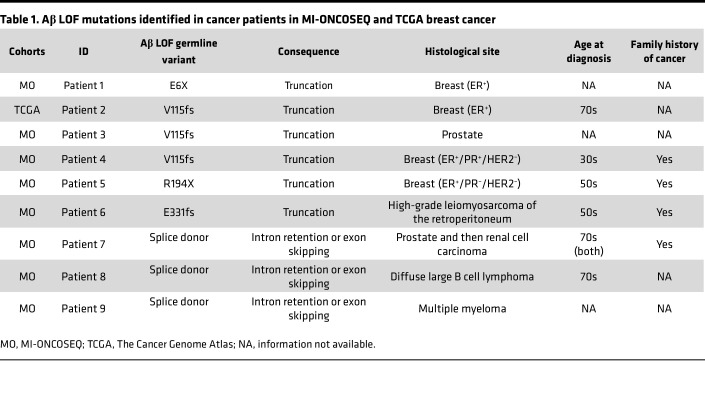
Aβ LOF mutations identified in cancer patients in MI-ONCOSEQ and TCGA breast cancer

## Version 1 04/03/2025

In-Press Preview

## Version 2 05/08/2025

Electronic publication
