# Supplementary material for: Germline mutations in PPP2R1B in patients with a personal and family history of cancer
Source: JCI Insight. 2025 Apr 3;10(9):e186288. doi: 10.1172/jci.insight.186288 (PMC12129040; doi:10.1172/jci.insight.186288)
Supplement: Supplemental data [file jciinsight-10-186288-s158.pdf]

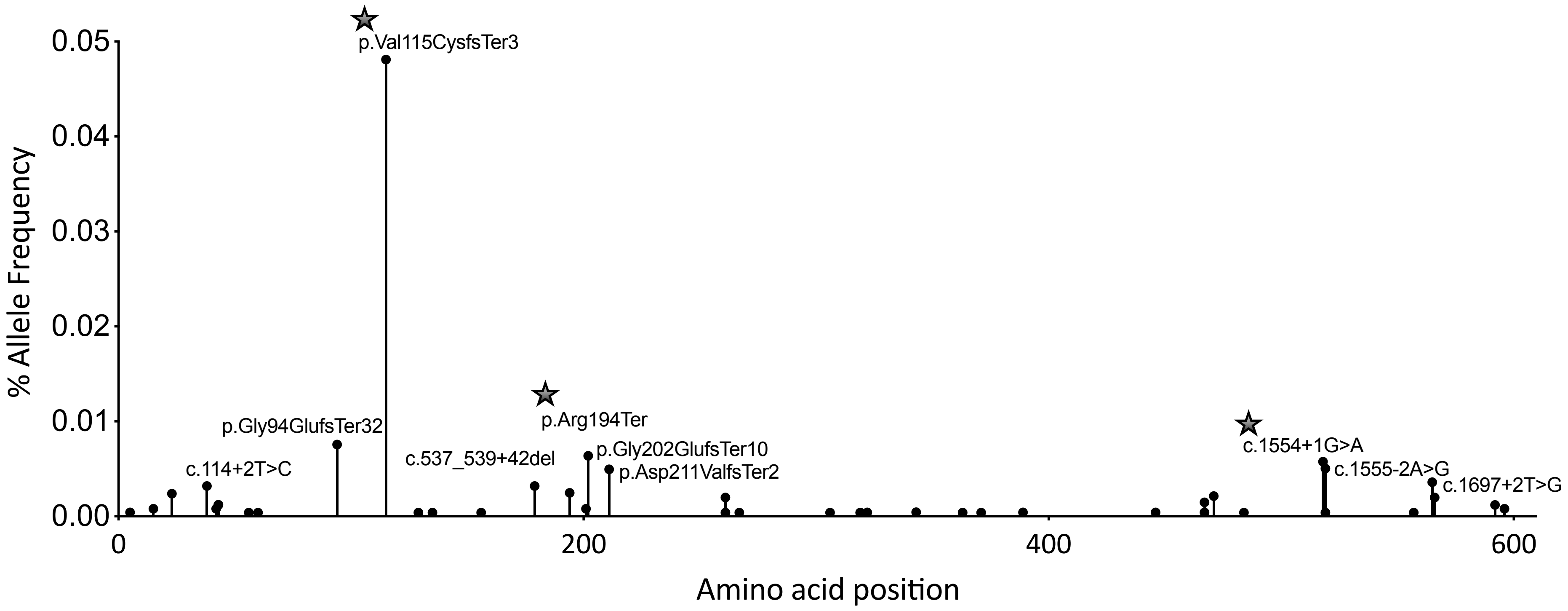

Supplementary Figure 1. All Aβ germline variants in gnomAD annotated as loss-of-function (high confidence). Y-axis represents percent allele frequency across all ethnicities and x-axis represents the position of the variants along Aβ. The 10 most frequent LOF variants are labelled. Stars indicate variants that were identified in cancer patients this study.

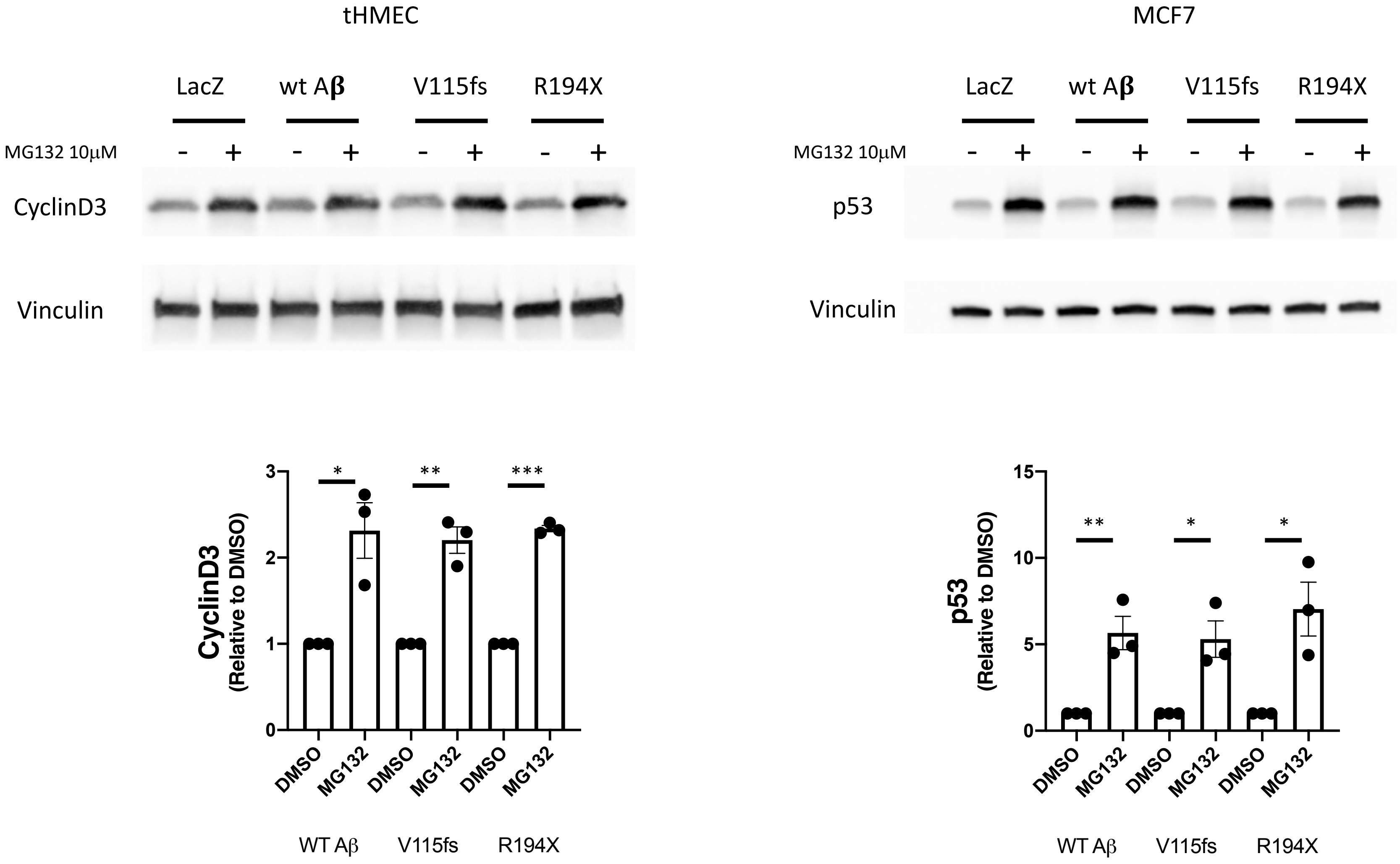

Supplementary Figure 2. Westerns blots and quantitation for CyclinD3 and p53 in tHMEC and MCF7 respectively in cells expressing wild type or truncated Aβ treated with MG132. Bars are Mean± SEM. * $p<0.05$, ** $p<0.01$, *** $p<0.001$

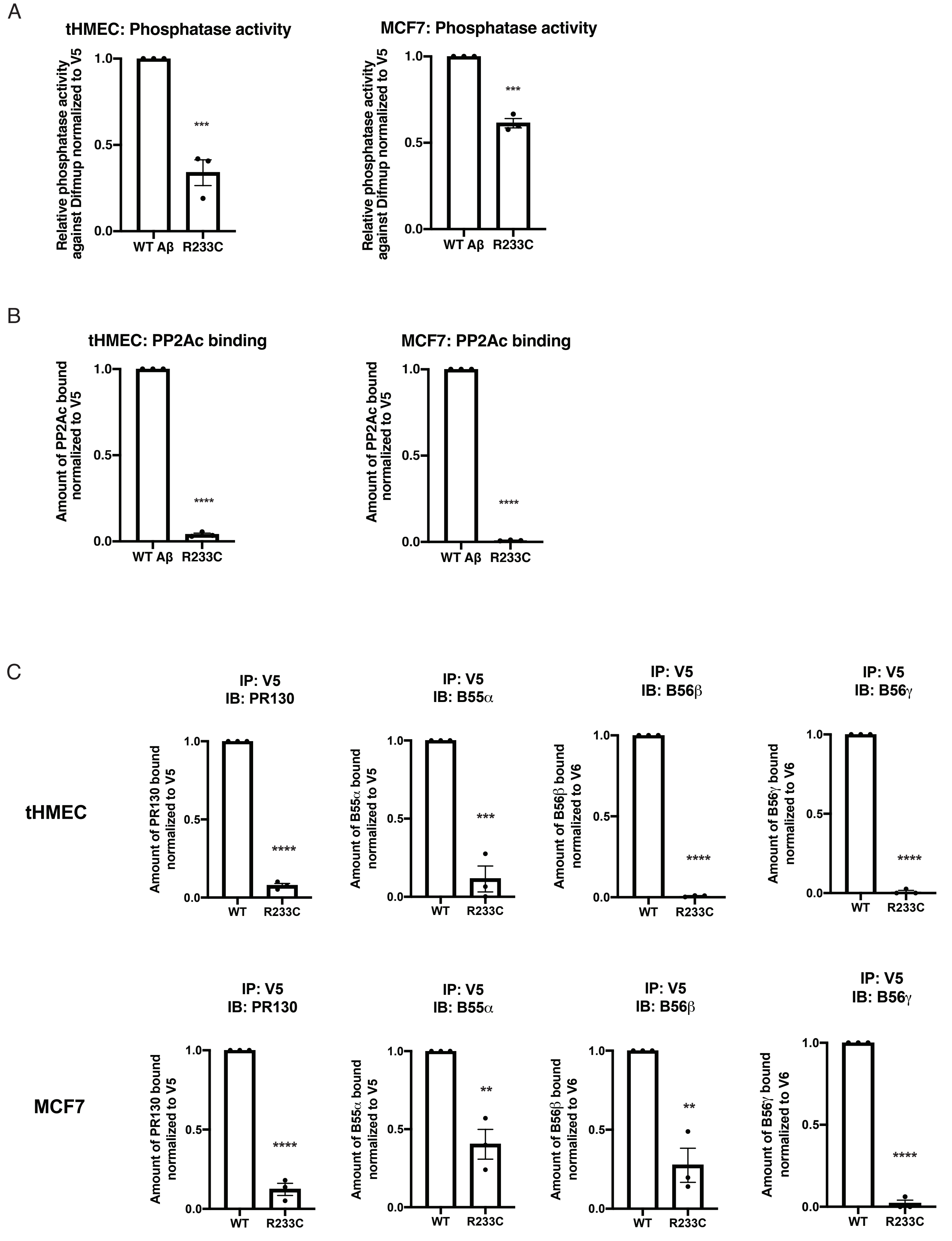

Supplementary Figure 3. R233C causes loss of catalytic activity and holoenzyme assembly. V5-tagged Wild type Aβ and R233C-Aβ were immunoprecipitated and the phosphatase activity of the CoIP material was determined. A) Quantitation of phosphatase activity against 400mM Difmup. B) Quantitation of PP2Ac (catalytic subunit) binding to wild type or R233C Ab. C) Quantitation of PP2A regulatory subunit binding to wild type or R233C Aβ. Bars are Mean± SEM. **p<0.01, ***p<0.001, ****p<0.0001.

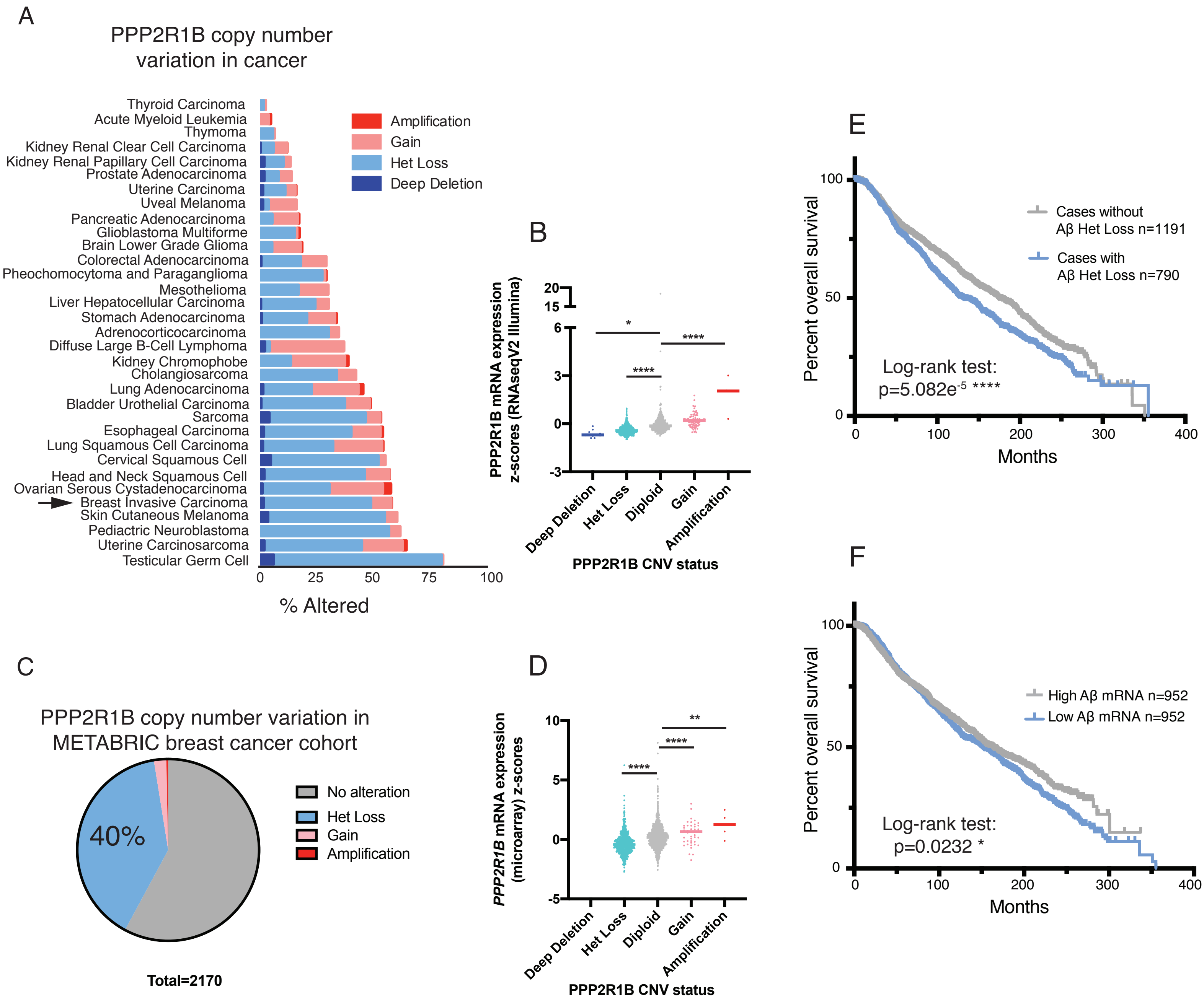

Supplementary Figure 4. Aβ heterozygous loss and decreased mRNA expression is a common event in breast cancer and correlates with reduced overall survival. A) Somatic copy number variation (CNV) in Aβ across cancer subtypes in the TCGA. B) Aβ CNV is significantly associated with mRNA expression in the breast cancer cohort of TCGA. C) Aβ CNV in the METABRIC cohort. D) Aβ CNV is significantly associated with mRNA expression in the METABRIC cohort. Significance calculated using One-way ANOVA with multiple comparisons, *<0.05, **<0.01, ***<0.001, ****<0.0001. E) Overall survival in breast cancer patients from the METABRIC cohort with heterozygous loss of Aβ. F) Overall survival in breast cancer patients from the METABRIC cohort with decreased Aβ mRNA. Cohort was dichotomized at the median. Data queried from cbioportal.org

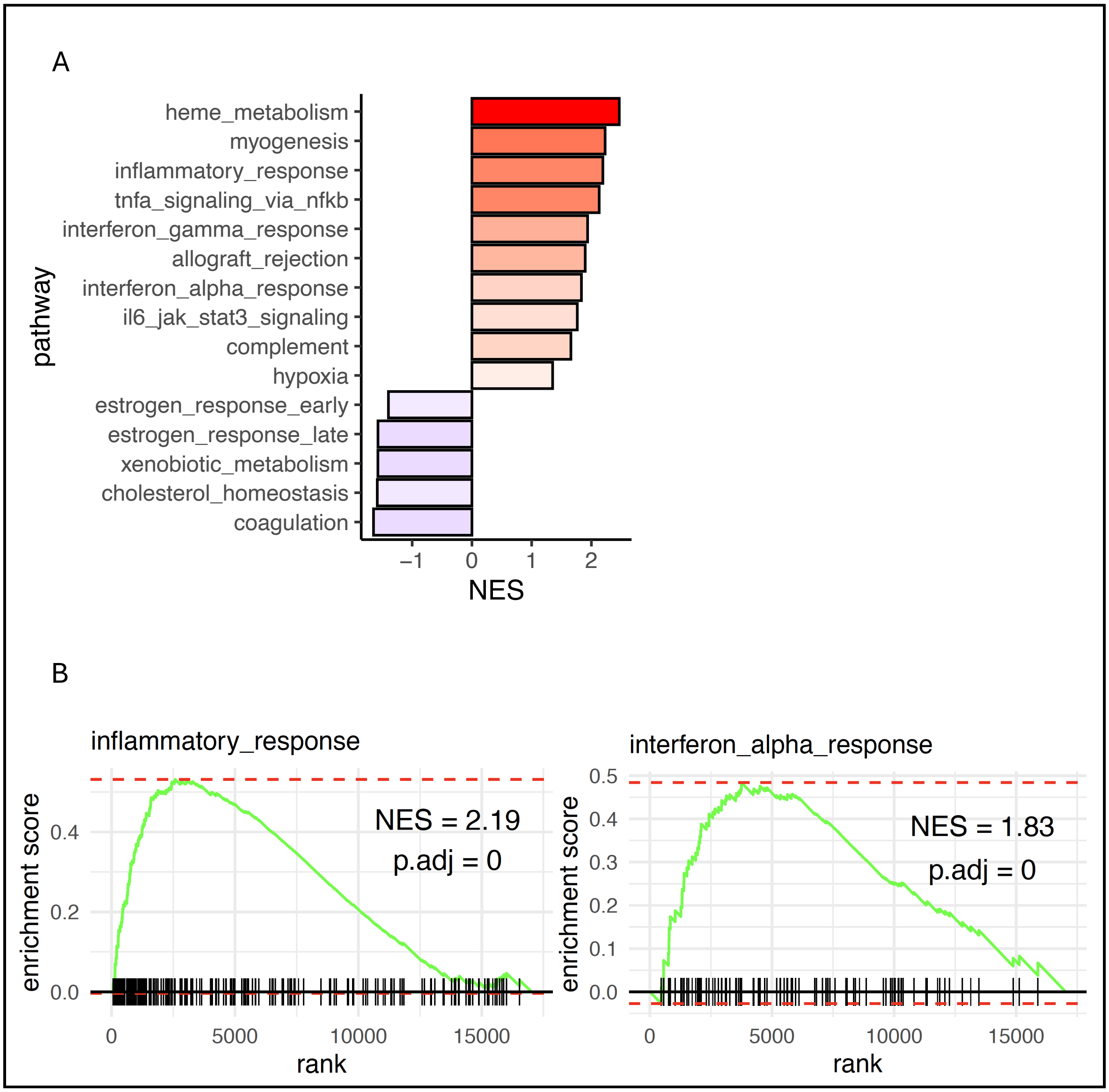

Supplementary Figure 5. (a) Significant hallmark pathways (FDR<0.05) in tumors with PPP2R1B germline mutations compared to tumors with wild type PPP2R1B (n=16); red indicates positive enrichment and blue indicates negative enrichment. (b) Enrichment plots of inflammatory response and interferon alpha response pathways.

Supplementary Table 1: Population data queried from gnomAD.org v2.1.1 (non-cancer) (134,187 samples). A value of “0” means that the allele was not found in any individual.

Protein Change	Exon	Transcript Consequence	Allele Frequency					
			African/African American	European (Finnish)	European (non-Finnish)	East Asian	South Asian	Others
**E6X**	1	c.16G>T	0	0	0	0	0	0
**V115fs**	4	c.343_344delGT	4.24E-05	2.79E-04	9.74E-04	0	0	1.16E-04
**R194X**	5	c.580C>T	0	3.98E-05	4.23E-05	0	0	0
**E331fs**	8	c.991_994delGAGA	0	0	0	0	0	0
**Splice donor lost**	12	c.1554+1G>A	4.24E-05	0	2.57E-05	0	0	2.40E-04

Supplementary Table 2: Germline variants in known predisposition genes.

Cohort		Aβ LOF germline variant	Histological site	BRCA1	BRCA2	CHEK2	PALB2	ATM	TP53	STK11	PTEN
MO	Patient #1	E6X	Breast (ER+)	none	none	none	none	none	none	none	none
TCGA	Patient #2	V115fs	Breast (ER+)	NA	NA	NA	NA	NA	NA	NA	NA
MO	Patient #3	V115fs	Prostate	none	none	none	none	none	none	none	none
MO	Patient #4	V115fs	Breast (ER+/PR+/HER-2-)	none	A2951T (benign)	none	none	none	none	none	none
MO	Patient #5	R194X	Breast (ER+/PR-/HER2-)	none	none	none	none	none	none	none	none
MO	Patient #6	E331fs	High-grade leiomyosarcoma of the retroperitoneum	none	none	none	none	none	none	none	none
MO	Patient #7	Splice donor	Prostate adenocarcinoma then renal cell carcinoma	none	none	none	none	none	N235S (likely benign)	none	none
MO	Patient #8	Splice donor	Diffuse large B-cell lymphoma	none	none	none	none	S978P (likely benign)	none	none	none
MO	Patient #9	Splice donor	Multiple Myeloma	G535E (no ClinVar record)	none	none	none	F582L (likely benign)	none	none	none

Variant classification is indicated in parentheses and was obtained from ClinVar. NA: Not available

Supplementary Table 3: Aβ tumor variant allele frequency and mRNA levels in cancer patients in Mi-Oncoseq (MO) and the breast cancer cohort of The Cancer Genome Atlas (TCGA) with LOF germline Aβ variants

Cohort		Aβ LOF germline variant	Histological site	Tumor variant allele frequency	Tumor Aβ mRNA Percentile
MO	Patient #1	E6X	Breast (ER+)	46%	high
TCGA	Patient #2	V115fs	Breast (ER+)	49%	low
MO	Patient #3	V115fs	Prostate	43%	high
MO	Patient #4	V115fs	Breast (ER+/PR+/HER-2-)	16%	normal
MO	Patient #5	R194X	Breast (ER+/PR-/HER2-)	65%	low
MO	Patient #6	E331fs	High-grade leiomyosarcoma of the retroperitoneum	11%	low
MO	Patient #7	Splice donor	Prostate adenocarcinoma then renal cell carcinoma	44%	normal
MO	Patient #8	Splice donor	Diffuse large B-cell lymphoma	43%	normal
MO	Patient #9	Splice donor	Multiple Myeloma	57%	normal

mRNA Percentile: >75% high; 25% -75% normal; <25% low

Supplementary Table 4: Recurrent R233C/H/L somatic mutation in multiple cancer types.

Cancer Type	Gene	Protein Change
Lung Squamous Cell Carcinoma	PPP2R1B	R233C
Pancreatic Adenocarcinoma	PPP2R1B	R233C
Uterine Endometrioid Carcinoma	PPP2R1B	R233C
Colorectal Adenocarcinoma	PPP2R1B	R233C
Colorectal Adenocarcinoma	PPP2R1B	R233L
Colorectal Adenocarcinoma	PPP2R1B	R233H
Glioblastoma Multiforme	PPP2R1B	R233H

Supplementary Table 5: Clinical parameters and *PPP2R1B* expression in lymph-node negative patients that did not receive any systemic adjuvant therapy. All samples had ≥30% tumor cells. Parameters with significant associations with *PPP2R1B* expression (p<0.05) are indicated in bold.

			PPP2R1B (log transformed)		
Clinical Parameters		Count	Median	IQR [25-75 percentile]	p value
All		830	-4.11	0.58	
ESR1	ESR1 negative<br>ESR1 positive	221<br>609	-4.11<br>-4.11	0.59<br>0.55	0.11•
PGR	PGR negative<br>PGR positive	326<br>504	-4.15<br>-4.08	0.57<br>0.57	**0.0003•**
ERBB2	ERBB2 unamplified<br>ERBB2 amplified	708<br>119	-4.15<br>-4.01	0.59<br>0.53	**0.001^**
Age at primary surgery	<=40<br>>40 - <=50<br>>50 - <=70<br>>70	107<br>204<br>369<br>150	-4.24<br>-4.09<br>-4.10<br>-4.10	0.59<br>0.51<br>0.61<br>0.52	0.51•
Menopausal status at primary surgery	pre-menopausal<br>post-menopausal	341<br>486	-4.11<br>-4.13	0.55<br>0.60	0.94^
T-status (Tumor size)	pT1; <=2 cm<br>pT2; >2 cm & <=5 cm<br>pT3; >5 cm	377<br>412<br>25	-4.08<br>-4.15<br>-4.03	0.60<br>0.59<br>0.58	**0.025♦**
Grade	poor<br>unknown<br>moderate/good	430<br>230<br>170	-4.17<br>-4.05<br>-4.07	0.60<br>0.53<br>0.58	**0.010♦**
Disease-free survival	=<1 year disease-free<br>1-3 years disease-free<br>>3 years disease-free	63<br>180<br>587	-4.12<br>-4.15<br>-4.09	0.59<br>0.49<br>0.60	**0.033♦**

ESR1: Estrogen Receptor; PGR: Progesterone receptor; ERBB2: Receptor tyrosine-protein kinase erbB-2 (HER2); Statistical tests used: • Spearman, ^ Mann-Whitney, ♦ Kruskal Wallis.
