# Supplementary material for: Germline mutations in PPP2R1B in patients with a personal and family history of cancer
Source: JCI Insight. 2025 Apr 3;10(9):e186288. doi: 10.1172/jci.insight.186288 (PMC12129040; doi:10.1172/jci.insight.186288)

Uncropped Westerns

Figure 2C

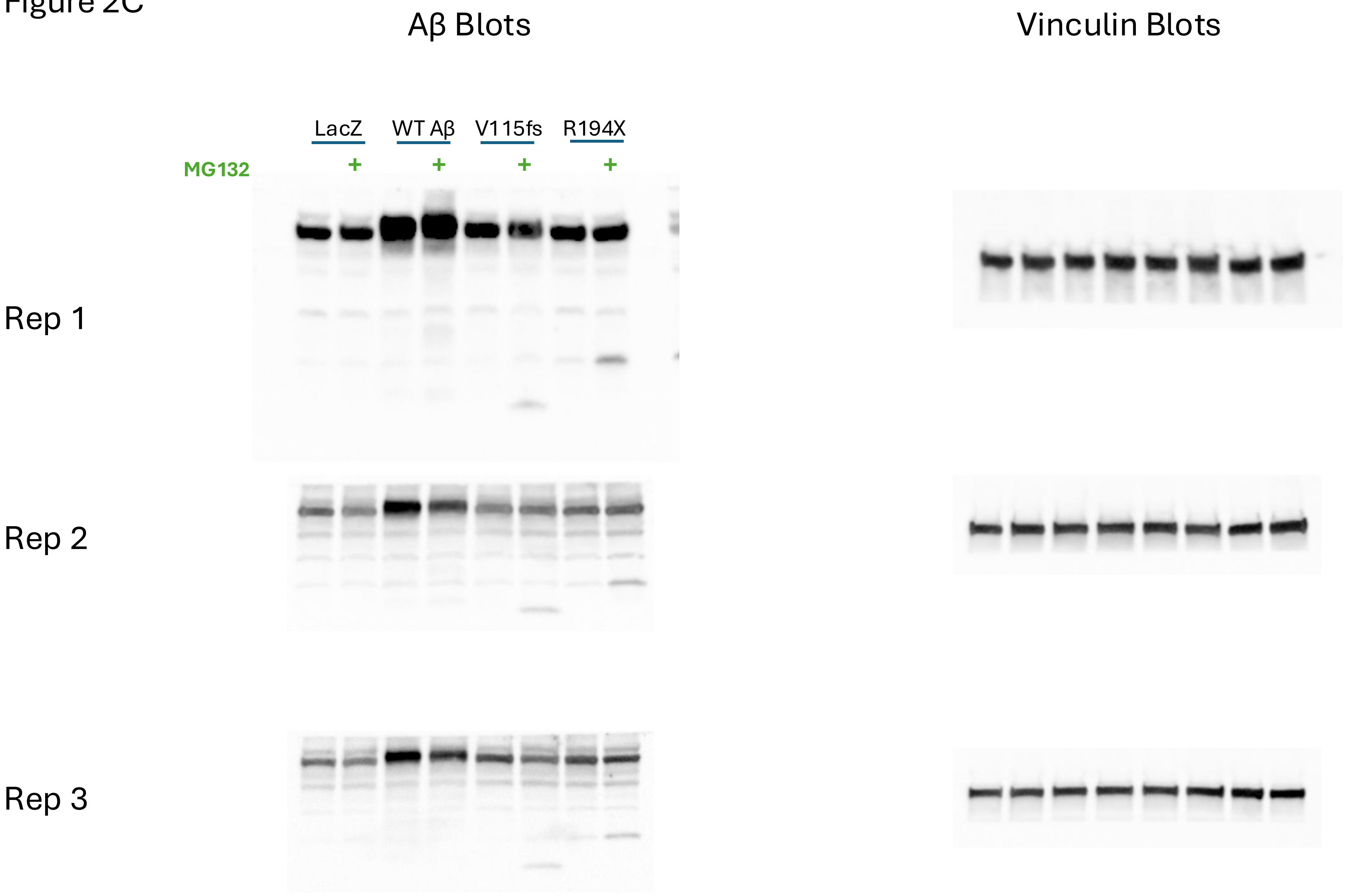

The full blot was cut and probed with anti-Vinculin or anti-Aβ

Figure 2D

* other condition that was not included in the manuscript

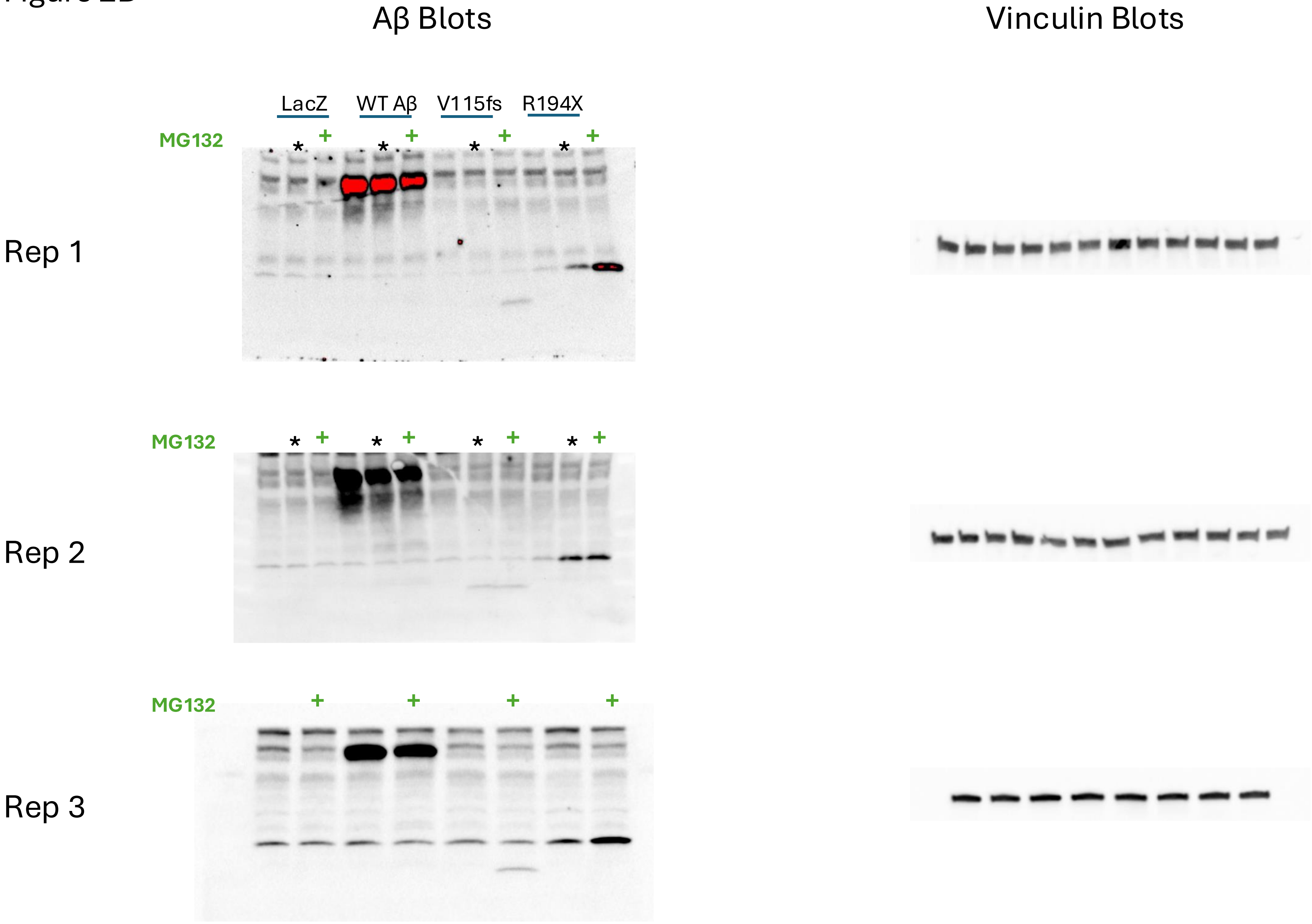

The full blot was cut and probed with anti-Vinculin or anti-Aβ

Figure 3B

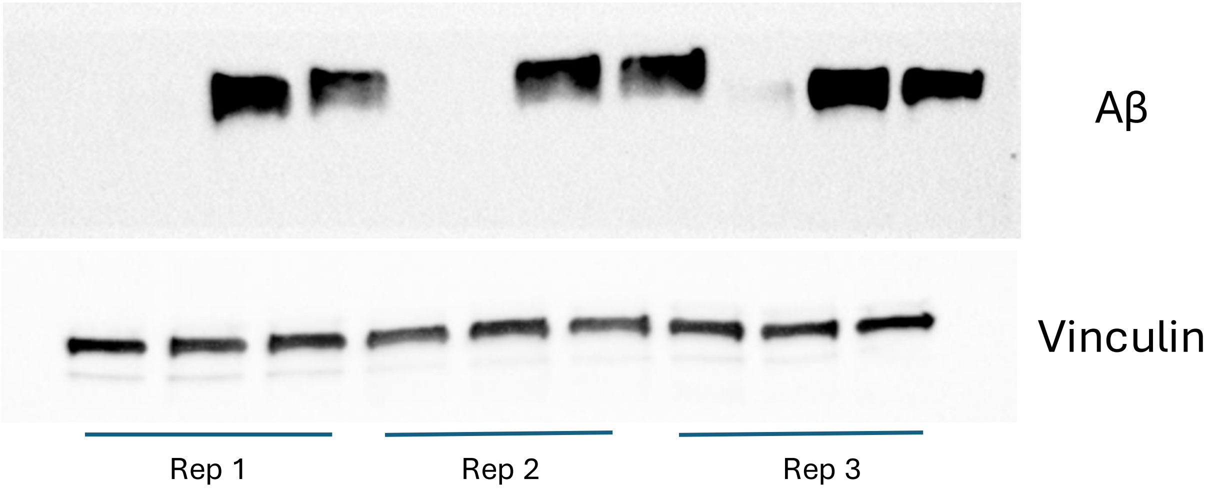

* other mutant that was not included in the manuscript

Figure 3C

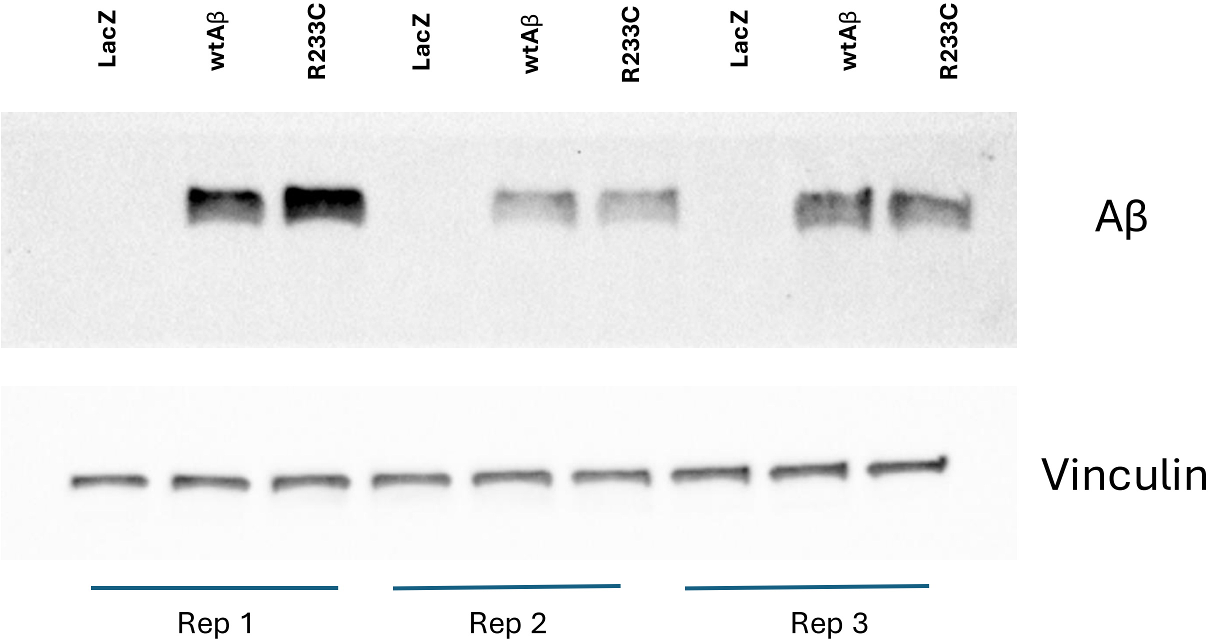

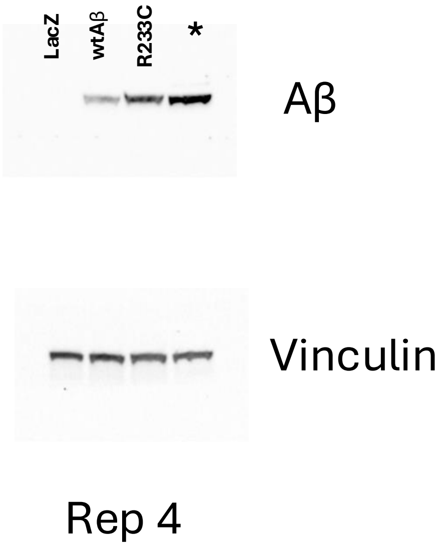

Figure 3F and 3H (tHMEC) INPUT (whole cell lysate)

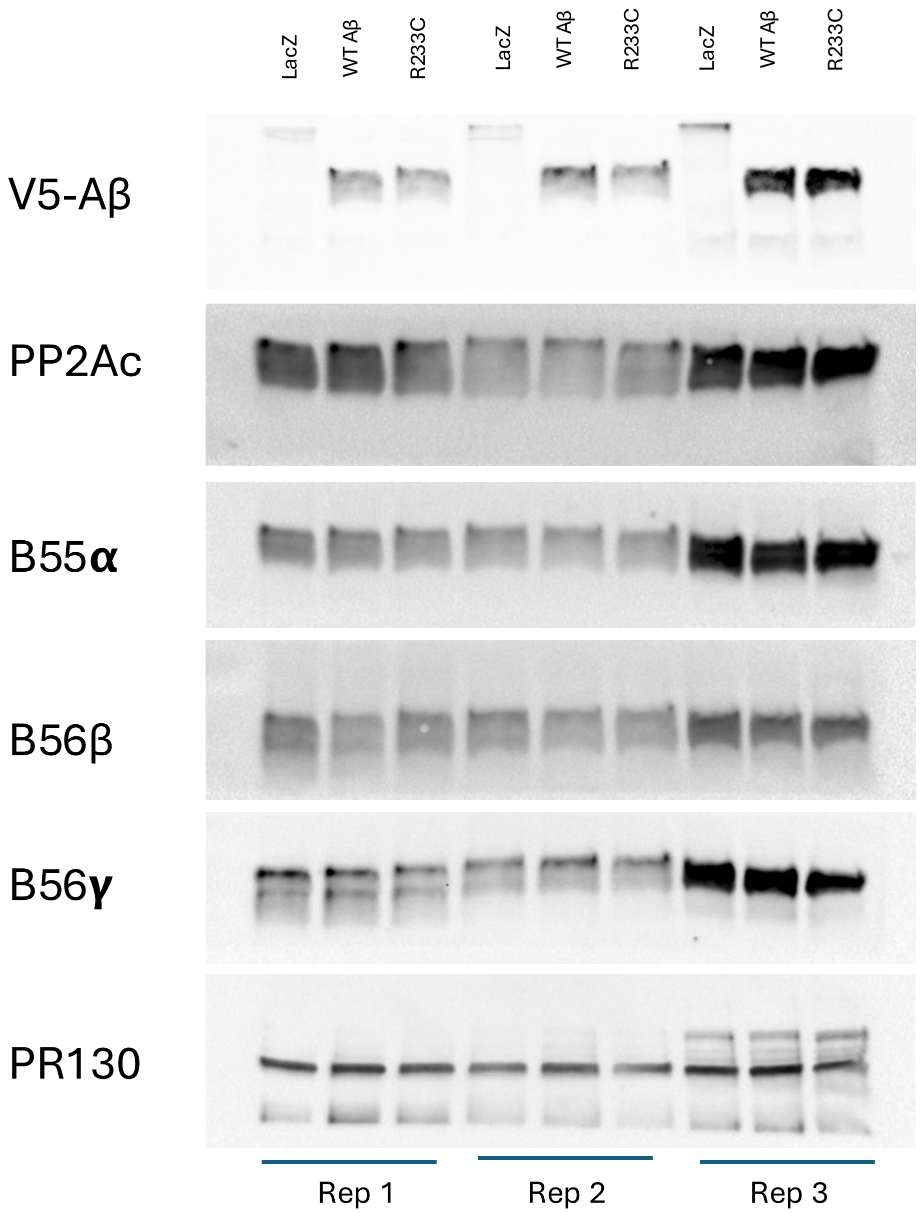

Figure 3G and 3I (MCF7) INPUT (Whole cell lysate)

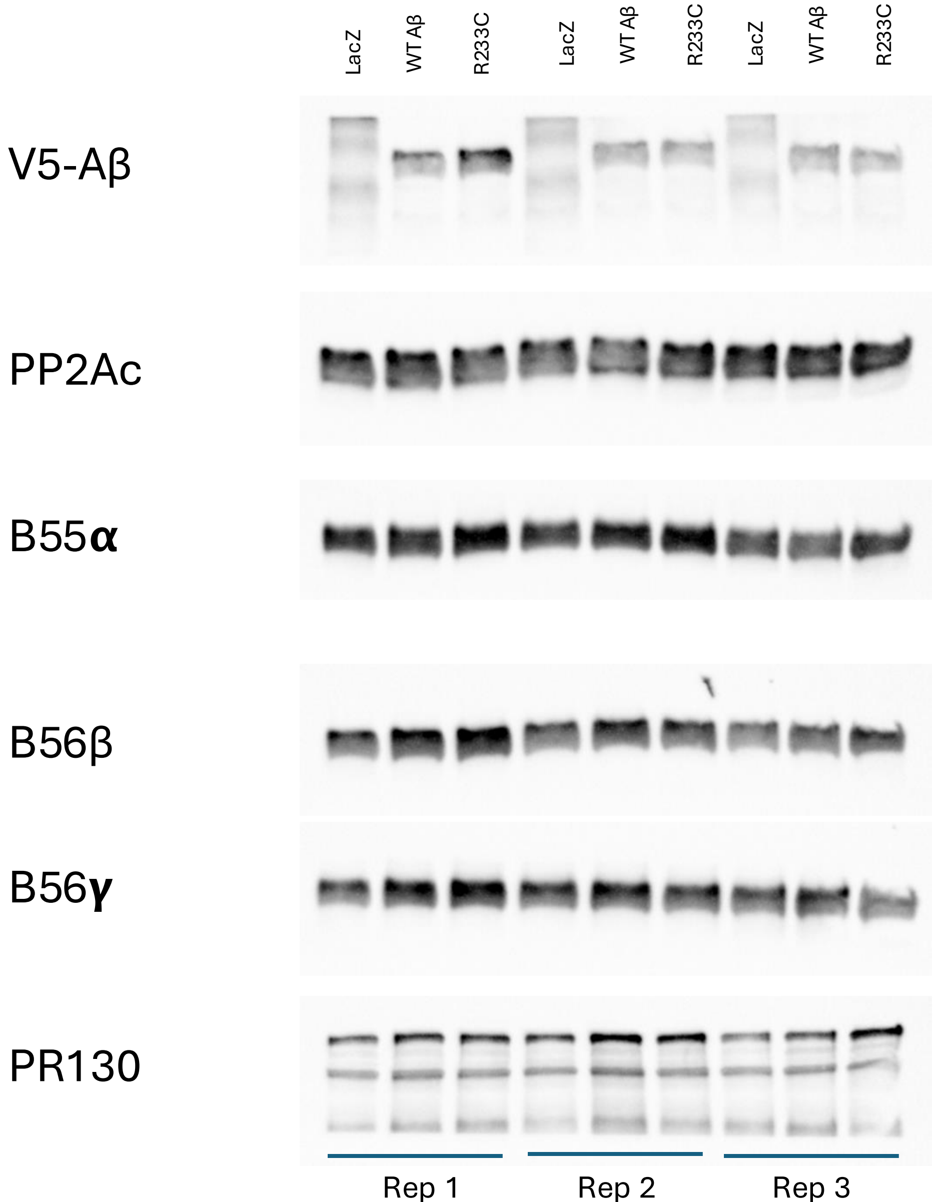

Figure 3F and 3H (tHMEC) CoIPs

* other mutant that was not included in the manuscript

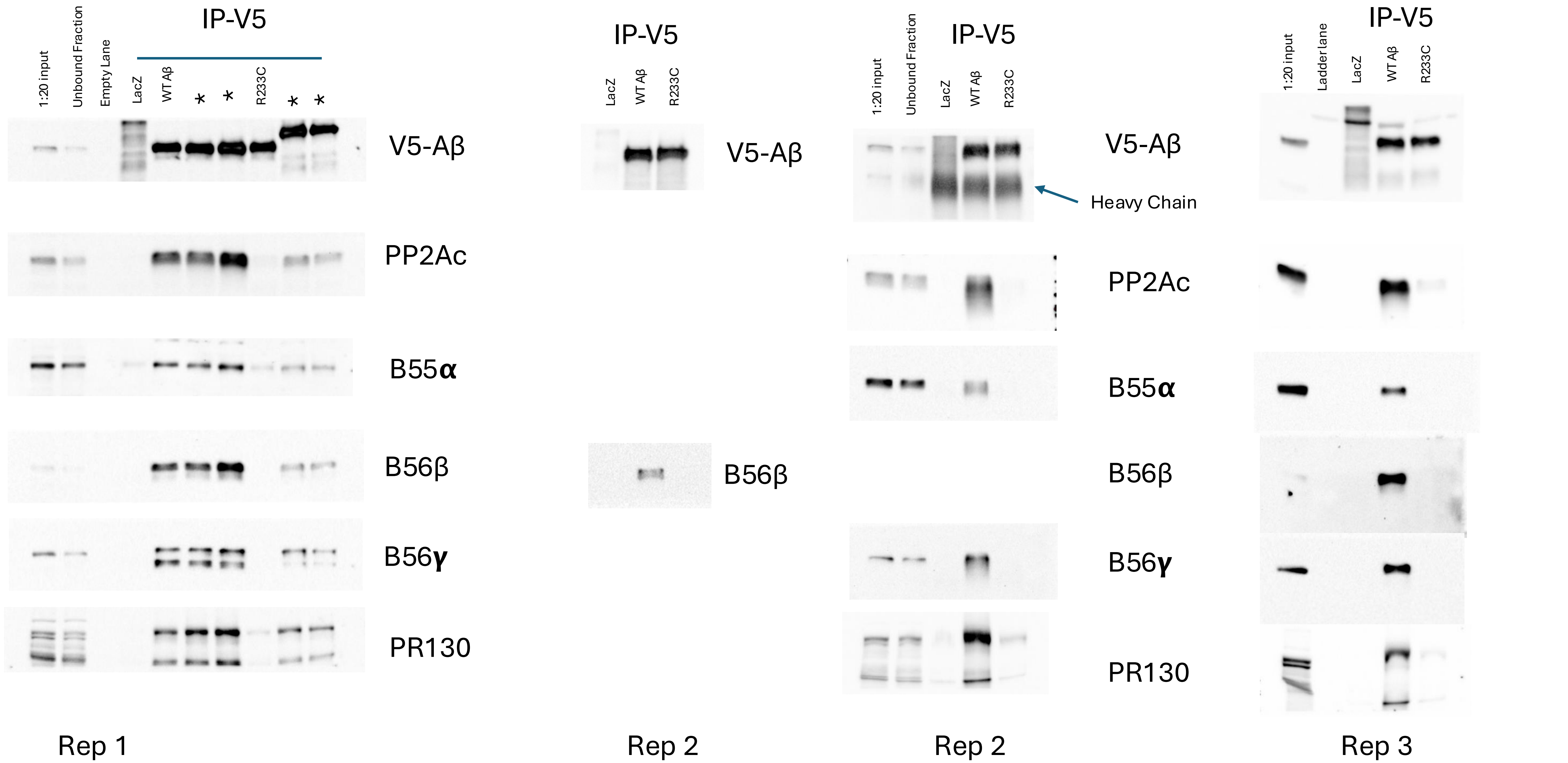

Figure 3G and 3I (MCF7) CoIPs

* other mutant that was not included in the manuscript

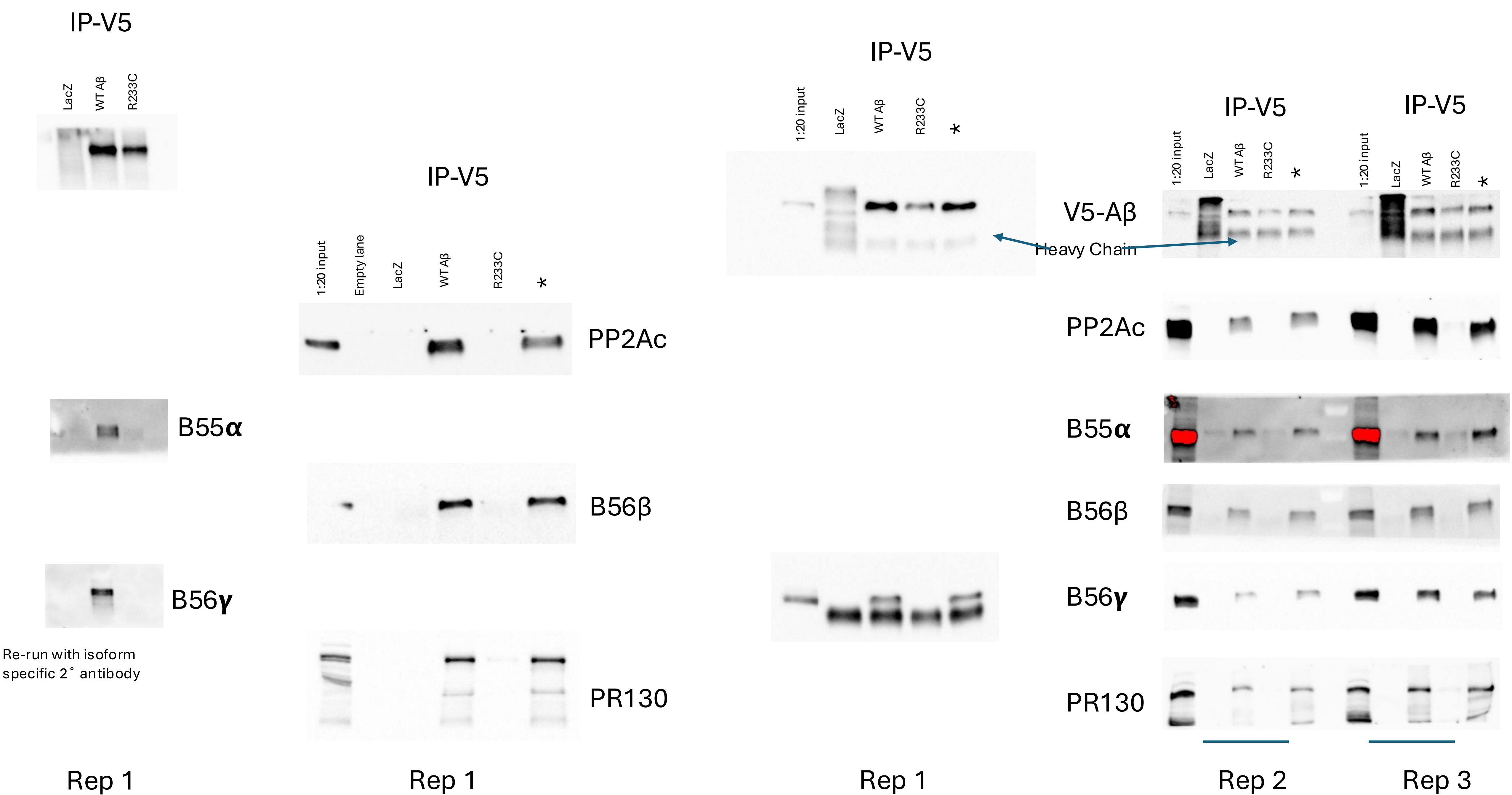

Supplement: Unedited blot and gel images [file jciinsight-10-186288-s159.pdf]
